# Prevention of postoperative nausea and vomiting after gynaecological day surgery under remimazolam general anesthesia: a randomized double-blind controlled study

**DOI:** 10.1186/s12871-022-01835-x

**Published:** 2022-09-15

**Authors:** Fuxia Yi, Hongyi Xiao, Teng Zhu, Yan Man, Fanceng Ji

**Affiliations:** 1 Department of Anesthesiology, Weifang People’ S Hospital, No. 151 Guangwen Street, Kuiwen District, Weifang, 261041 China; 2 School of Anesthesiology, Weifang Medical University, Weifang, China

**Keywords:** PONV, Remimazolam, Alfentanil, Dexamethasone, Droperidol, Tropisetron

## Abstract

**Purpose:**

To observe the effect of different antiemetic drugs for the prevention of postoperative nausea and vomiting (PONV) after gynaecological day surgery under remimazolam general anesthesia.

**Methods:**

One hundred ninety-two patients were selected for gynaecological day surgery and randomly divided into three groups: droperidol group (DD group), tropisetron group (DT group) and control group (DC group). Flurbiprofen axetil 50 mg and dexamethasone 5 mg were given intravenously before induction of anesthesia, and 2 min later droperidol 1 mg was given intravenously to the DD group, tropisetron 5 mg to the DT group and saline (5 ml) to the DC group. Induction of anesthesia: remimazolam 6 mg/kg/h was continuously infused until sleep, mivacurium 0.2 mg/kg and alfentanil 20ug/kg were slowly pushed, 3 min later intubation was performed to control breathing. Maintenance of anesthesia: 40ug/kg/h of alfentanil, 1 mg/kg/h of remimazolam continuous infusion. After awakening and extubation, the patient was transferred to the PACU. PONV were recorded in the PACU and an electronic questionnaire was pushed 24 h after surgery.

**Results:**

The incidence of PONV within the PACU was significantly lower in the DD (14.5%)and DT(26.7%) groups than in the DC(50%) group (*p* < 0.01), there was no significantly difference between the DT and DD groups. There were no significant difference in the incidence of PONV in 24 h after surgery between the three groups(DD:DT:DC = 44.5%:45.1%:63.8%,*p* > 0.05).

**Conclusions:**

Droperidol or tropisetron combined with dexamethasone is superior to dexamethasone alone for the prevention of PONV in the PACU after remimazolam combined with alfentanil anesthesia, with no significant difference in the incidence of PONV in 24 h after surgery.

## Introduction

Postoperative nausea and vomiting (PONV) is one of the most common complications of general anesthesia [1]. The risk factors of PONV include the patient-related factors, anesthetic factors and surgical factors. Gynecological day surgery patients are at high risk for PONV in terms of gender, age, motion sickness, gynecological surgery and opioids, with PONV being as high as 80% in high-risk patients.

Remimazolam is a novel benzodiazepine that acts on central GABA_A_ receptors to produce sedation and amnesia and is widely used for preoperative administration, endoscopic anesthesia, induction maintenance of general anesthesia and in ICU administrations [2]. Alfentanil is a short-acting opioid with low respiratory depression, less cough induced and fast metabolism, which is suitable for daytime surgical anesthesia. The combination of different types of antiemetic drugs is better than single drugs for prevention and treatment, and reduces side effects [3]. 5-HT_3_ receptor inhibitors, dexamethasone and droperidol are commonly used for the prevention of PONV. Whether the combination of the above drugs is effective in preventing PONV after remimazolam combined with alfentanil total intravenous anesthesia deserves clinical investigation.

## Materials and methods

### Patients and study protocol

The study was approved by the Ethics Committee of Weifang People’s Hospital and registered with the China Clinical Trials Registry (ChiCTR2100053316). The subjects was 192 patients of ASA1-2 level, aged between 18 and 65 years, who were about to undergo gynaecological day surgery at the First Clinical Medical College of Weifang Medical College. Exclusion criteria were breastfeeding, a history of chronic pain, a history of sedative and analgesic administration or allergy to any of the study drugs, severe hypertension, and diabetes mellitus. Reject criteria were a procedure time of more than 1 h, discharged the next day, missing follow-up with the electronic questionnaire pushed 24 h after the procedure. Randomly divided into 3 groups: DD group (dexamethasone combined with droperidol group), DT group (dexamethasone combined with Tropisetron group) and DC group (dexamethasone group). The computer-generated random allocation sequence was created by an independent investigator using Excel 2016 (Microsoft) with a 1:1:1 allocation and random block sizes. On the morning of the surgery, the anesthesia nurse opened the envelope containing the anesthesia scheme of the enrolled patients and then prepared the drugs. Each drug was diluted to 10 ml with normal saline, and the drug type was identified without the drug name. Participants and outcome assessors were blinded to group allocation.

Patients were assessed by the anesthesia clinic before admission and confirmed to be ready for day surgery. On the day of surgery, patients were admitted to the room and re-confirmed to be free of contra-indications and then intravenous access was established, lactated ringer’s solution was infused and NIBP, HR and SPO_2_ were monitored. flurbiprofen axetil 50 mg and dexamethasone 5 mg were given intravenously before induction of anesthesia and 2 min later droperidol 1 mg was given to the DD group, tropisetron 5 mg to the DT group and saline (5 ml) to the DC group. Induction of anesthesia: remimazolam 6 mg/kg/h was continuously infused until sleep, mivacurium 0.2 mg/kg and alfentanil 20ug/kg were slowly injected, 3 min later tracheal intubation was performed to control breathing. Anesthesia maintenance: alfentanil 40ug/kg/h and remimazolam 1 mg/kg/h continuous infusion, stop infusion at the end of the operation. A single dose of remimazolam 2 mg or and alfentanil 80ug was given at the onset of intraoperative signs of decompensated anesthesia. After awakening and extubation, the patient was taken to the PACU and assessed for nausea and vomiting. Patients were discharged after meeting discharge criteria as assessed by the Post-anesthetic Discharge Scoring System (PADSS) criteria. An electronic follow-up questionnaire was pushed 24 h after the operation. Basic information about the patient’s medical history and surgery was obtained through preoperative anesthesia clinic assessment, intraoperative anesthesia monitoring, in the inpatient electronic medical record, and observation notes in the PACU.

### Main outcome

The incidence of PONV in the PACU.

### Secondary outcome

The incidence of PONV within 24 h after surgery; Baseline data on age, height, weight, BMI, ASA grading, Apfel score, type of surgery and general data such as remimazolam and alfentanil consumption and duration of surgery.

### Sample size and statistical analysis

There are no reports of PONV after general anesthesia with remimazolam combined with alfentanil. According to the literature[4, 5], we applied intravenous general anesthesia with remimazolam combined with alfentanil in gynaecological day surgery, and the anesthetic effect was good; in this study, α = 0.05 and β = 0.1 were taken and the results of the pre-test study were that the incidence PONV in the PACU was approximately 55% in the DC group, 25% in the DD group and 15% in the DT group. The sample size with statistically significant differences between the DC and DD groups was calculated to be 57 cases, with a 10% missing sample rate, so 64 cases were included in each group, for a total of 192 cases in the three groups..

SPSS 18.0 software was used for statistical analysis, and measurement data that obeyed normal distribution were expressed as mean ± standard deviation (x ± s)and compared using analysis of variance (ANOVA). Non-parametric rank sum test was used for non-normally distributed measurement data. Count data were expressed as rates or composition ratios, and the χ2 test was used. A *P* value of < 0.05 was considered to be statistically significant.

## Results

**Fig. 1:**
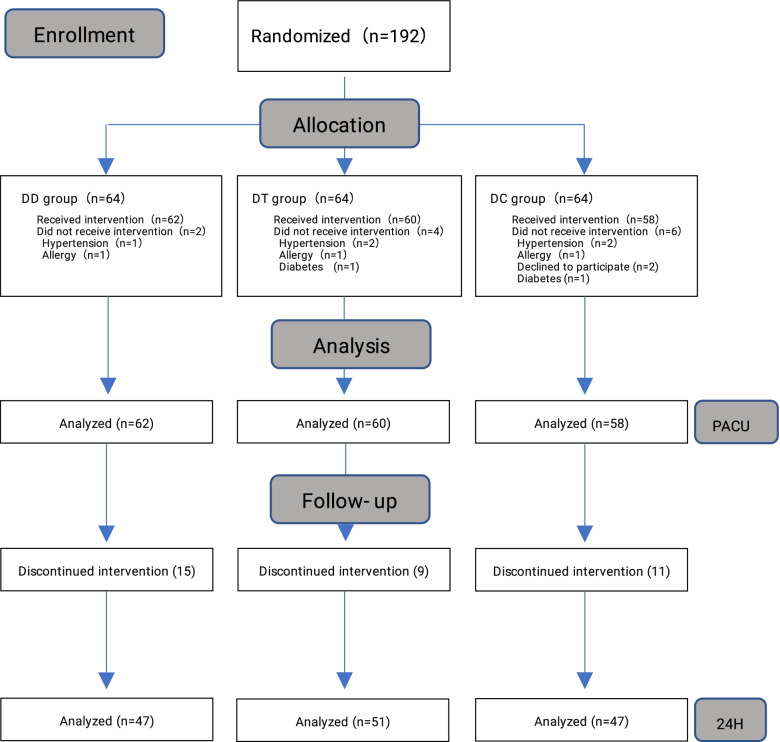
A total number of 192 patients were randomized into three groups (*n* = 64). For various reasons (Hypertension, Allergy, Diabetes or Declined to participate), 2 patients in DD the group, 4 patients in the DT group and 6 patients in the DC group did not receive trial medication. Thus 180 patients were analyzed: 62 patients in the DD group,60 patients in the DT group, 58 patients in the DC group. For 35 patients lost to follow up after discharge, 145 patients were analyzed: 47 patients in the DD group, 51 patients in the DT group, 47 patients in the DC group

One hundred ninety-two patients were included, six were excluded and six were rejected, and data from 180 patients in the PACU and 145 patients at 24 h postoperatively were obtained for statistical analysis (Fig. 1).

There were no significantly differences between the three groups in the basic data of patients such as age, weight, height, BMI, ASA grade, Apfel score, type of surgery and the dosage of remimazolam and alfentanil and duration of surgery (*P* > 0.05) (Table 1).

**Table 1:** Patient demographics

Characteristic	DD group (*n* = 62)	DT group (*n* = 60)	DC group (58)	F/χ2 /K-W	*P* value
Age, yr	43.41 ± 1.35	43.85 ± 1.15	43.63 ± 1.28	0.068(F)	0.934
Height, m	1.62 ± 0.01	1.62 ± 0.01	1.61 ± 0.01	0.357(F)	0.700
Weight, kg	61.80 ± 1.03	62.05 ± 1.30	60.82 ± 1.15	0.357(F)	0.700
BMI, kg/m^2^	23.59 ± 0.41	23.74 ± 0.46	23.46 ± 0.44	0.132(F)	0.876
ASA class n(%)					
I	8(12.9%)	14(23.3%)	12(20.7%)	2.346(χ2)	0.309
II	54(87.1%)	46(76.7%)	46(79.3%)		
Apfel score n(%)					
2	39(62.9%)	32(53.3%)	40(69.0%)	3.110(χ2)	0.211
3	23(37.1%)	28(46.7%)	18(31.0%)		
Type of surgery, n(%)					
Hysteroscope	46(75.4%)	40(66.7%)	44(75.9%)	1.698(χ2)	0.791
Cervical conization	14(23.0%)	19(31.7%)	13(22.4%)		
Others	1(1.6%)	1(1.7%)	1(1.7%)		
Duration of anesthesia, min Median (Range)	23.00(20.00–30.00)	24.50(16.00–36.75)	24.50(17.50–29.50)	0.009(K-W)	*P* = 0.996
Dose, mg					
Remimazolam, induction	16.38 ± 0.41	15.84 ± 0.48	15.31 ± 0.36	1.287(F)	0.279
Remimazolam, maintenance,Median (Range)	13.87(8.67–20.85)	14.80(9.70–20.75)	15.00(10.00–23.00)	0.771(K-W)	0.680
Alfentanil, induction,Median (Range)	1.00(1.00–1.00)	1.00(1.00–1.00)	1.00(1.00–1.10)	1.908(K-W)	0.385
Alfentanil, maintenance,Median (Range)	0.46(0.32–0.68)	0.57(0.37–1.06)	0.56(0.40–0.90)	4.749(K-W)	0.093
Mivacurium Chloride induction,Median (Range)	10.00(10.00–10.00)	10.00(10.00–10.00)	10.00(10.00–12.00)	1.834(K-W)	0.400

*ASA* American Society of Anesthesiologist, *BMI* Body Mass Index

Data are presented as mean ± SD, number of patients or Median (Range)

The incidence of PONV in the PACU was significantly lower in the DD(14.5%) and DT (26.7%)groups than in the DC(50%) group (*P* < 0.01) (Table 1), and the difference between the DD and DT groups was not significantly (*P* > 0.05) (Table 2).The incidence of PONV in 24 h after surgery was no significantly difference between the three groups (DD:DT:DC = 44.5%:45.1%:63.8%,*P* > 0.05) (Table 2).

**Table 2:** Postoperative values

PONV	DD group	DT group	DC group	F/χ2 /H(K)	*P* value
PACU(n)	62	60	58		
0	53(85.5%)	44(73.3%)	29(50.0%)	18.443	0.000
1	9(14.5%)	16(26.7%)	29(50.0%)^a^		
24 h(n)	47	51	47		
0	26(55.3%)	28(54.9%)	17(36.2%)	4.558	0.102
1	21(44.7%)	23(45.1%)	30(63.8%)		

*PONV* Postoperative nausea and vomiting *PACU* Postanethesia care unit

0 = no of nausea 1 = nausea or/and vomiting

^a^*P* < 0.01 for DC group vs DD group and DT group

## Discussion

Remimazolam is a new type of ultra-short-acting benzodiazepine that acts on the central GABA_A_ receptor, opening the channel and increasing the inward flow of chloride ions, causing hyperpolarization of the nerve cell membrane and thus inhibiting neuronal activity, producing sedation and amnesia etc. Remimazolam combines some of the properties of both remifentanil and midazolam. It is derived from the parent compound midazolam and incorporates the pharmacokinetic properties of remifentanil, which is metabolised by tissue esterases to an inactive compound CNS 7054. It has three times the total drug clearance of midazolam and is characterised by a rapid onset of action and mild respiratory and circulatory depression [6]. However, it does not have the analgesic effect of opioids and often needs to be used in combination with other opioid analgesics. Continuous infusion of 3 h of context-sensitive half-time (CSHT) is similar to that of propofol (7.5 min) and significantly shorter than that of midazolam [7]. There are specific antagonists for remimazolam [8]. In 2020, remimazolam was approved for induction and maintenance of general anesthesia in Japan [9] and in March 2021,remimazolam besylate was approved for induction and maintenance of general anesthesia in China. Studies have confirmed that remimazolam (0.2 mg/kg, 0.3 mg/kg, 0.4 mg/kg) is a safe and effective sedative drug with few side effects during induction of anesthesia in ASAI-II patients and provides stable hemodynamics compared to propofol [10]; remimazolam can be used safely and effectively instead of propofol for induction of anesthesia for valve replacement [11]. In this study, induction and maintenance of anesthesia was performed according to a regimen of 6 mg/kg/h initial infusion of remimazolam induction and 1 mg/kg/h maintenance [12, 13].

A large body of literature confirms that the incidence of PONV is approximately 25 ~ 50% [14] and that post-discharge nausea and vomiting (PDNV) occurs in 30% of patients [15]. Despite the widespread use of anti-emetic drugs, short-acting anesthetic drugs and minimally invasive surgery in the clinic, the incidence of PONV is still 20 ~ 40% [16, 17] and up to 80% in high-risk groups, mainly associated with increased day surgery and early activity and discharge after minor/major surgery [18].High risk factors for early PONV in the PACU are opioid administration, female gender, BMI > 35, major surgery, and duration of anesthesia over 60 min [19]. The Apfel score of the PONV risk scale is a better predictor of the risk of PONV occurrence. The Apfel score for PONV risk assessment in the three groups of patients in this study was 2–3, which is an intermediate to high risk of PONV. There were no statistically significant differences in basic information such as age, weight, BMI, ASA grade and type of surgery among the three groups of patients. There were no statistically significant differences in the intraoperative administration of alfentanil and remimazolam and the duration of surgery, so the baseline information of the three groups of patients was comparable.

The incidence of PONV in the PACU in this study was 14.5% in the dexamethasone combined with droperidol group, 26.7% in the dexamethasone combined with tropisetron group and 50% in the dexamethasone alone group. the incidence of PONV within 24 h was 44.7% in the dexamethasone combined with droperidol group, 45.1% in the dexamethasone combined with tropisetron group and 51% in the dexamethasone alone group. The incidence of PONV falls within the normal incidence range reported in the literature.

Dexamethasone is a corticosteroid antiemetic that is currently widely used in clinical practice and may be associated with anti-infective effects and stabilization of cell membranes. Dexamethasone 4-12 mg given intravenously was effective in preventing PONV [20]. Because of the slow onset of action of dexamethasone, this study followed the recommendation to administer it at the start of surgery. Perioperative use of dexamethasone did not reduce the incidence of PONV in the PACU in a multi-centre study, but did reduce PDNV [21]. 5-HT_3_ receptors are closely related to PONV and can act from the cerebral cortex, chemical trigger band, vomiting centre and visceral afferent nerves. Droperidol has strong Dopamine receptor antagonism, and low dose (0.625–1.25 mg) can effectively prevent PONV. Dexamethasone combined with ondansetron can effectively prevent early and late PONV [20]. The combination of different types of antiemetic drugs can block a variety of central nervous system receptors and have a better preventive effect than single drugs. Combination therapy is recommended to prevent PONV. The use of the least effective dose also reduced the incidence of side effects for each drug. Habib et al.found in a multi-centre randomized controlled trial that the combination of a 5-HT_3_ receptor antagonist and dexamethasone was significantly more effective than the 5-HT_3_ receptor antagonist alone in preventing PONV [22]. Dexamethasone, ondansetron and droperidol were used in combination at doses not exceeding 8 mg, 4 mg and 1.25 mg [23], and intravenous administration in this study was within the recommended dose. 5-HT_3_ combined with droperidol and droperidol combined with dexamethasone did not differ [22], and 5-HT_3_ receptor inhibitors, dexamethasone and droperidol were effective in preventing PONV with few side effects. This study confirmed that either droperidol or tropisetron combined with dexamethasone reduced the incidence of PONV in the PACU better than dexamethasone alone, but whether droperidol was superior to tropisetron combined with dexamethasone (26.7% & 14.5%) may not be statistically different due to the sample size. There is no conclusive evidence on the most optimal drug combination and dose selection [24].

This study confirmed that the incidence of PONV in the three groups was 44.7–63.8% within 24 h postoperatively, with no difference, which may be related to the short duration of the antiemetic effect of a single administration of droperidol or tropisetron, thus necessitating further research into postoperative interventions for PONV.

As to whether remimazolam itself has a preventive effect on PONV, it has been suggested in the literature that midazolam can reduce the incidence of PONV when applied either at the induction of anesthesia or at the end of surgery [25]; Midazolam 2 mg given 30 min before the end of surgery can effectively prevent PONV and is equivalent to ondansetron 4 mg. Hari Y et al. found that remimazolam reduced the incidence of early postoperative nausea and vomiting compared with desflurane in gynaecological laparoscopic surgery, with no difference for PONV 24 h after surgery [1].

The pathogenesis of PONV is complex, and understanding the risk factors of PONV, the effectiveness of various antiemetic drugs and non-pharmacological treatment countermeasures will enable us to further understand PONV, optimise anesthetic management methods to reduce the risk factors for PONV under the premise of meeting surgical needs, and use antiemetics early and appropriately for high-risk patients or supplement with other non-pharmacological treatments.

Inadequacies of this study: The three groups of patients with Apfel scores of 2–3 were at moderate to high risk of PONV, and for ethical reasons there was no blank control group, so it was not possible to determine whether dexamethasone alone could reduce the incidence of PONV after anesthesia for this type of procedure. For the incidence of PONV within 24 h being significantly higher than the incidence of PONV in the PACU, are there any factors related to the patient’s change in position and premature discharge activity? As we found that patients in the PACU were prone to PONV during position change. No combination of the three drugs was taken for high-risk patients., and 5-HT_3_ receptor inhibitors have been shown to work best in combination with droperidol and dexamethasone. The gold standard for determining the effectiveness of clinical control of PONV is to achieve 24 h effectiveness and complete absence of nausea and vomiting, so it is important to choose antiemetic drugs and timing of administration appropriately. This study protocol did not achieve effective prevention of PONV at 24 h and further optimization of the protocol is required. It is also uncertain whether the short-acting benzodiazepine remimazolam also has a preventive effect against PONV in this study. Further studies are therefore needed for the postoperative PONV of remimazolam and the interaction with opioids [26].

In conclusion, droperidol or tropisetron combined with dexamethasone was effective in reducing the incidence of PONV in the remimazolam combined with alfentanil PACU compared to dexamethasone alone, but had no effect on the incidence of PONV in 24 h after surgery.

## Data Availability

The datasets generated and analysed during the current study are not publicly available due to institutional restrictions but are available from the corresponding author on reasonable request.
